# Session-by-Session Prediction of Anti-Endothelial Growth Factor Injection Needs in Neovascular Age-Related Macular Degeneration Using Optical-Coherence-Tomography-Derived Features and Machine Learning

**DOI:** 10.3390/diagnostics14232609

**Published:** 2024-11-21

**Authors:** Flavio Ragni, Stefano Bovo, Andrea Zen, Diego Sona, Katia De Nadai, Ginevra Giovanna Adamo, Marco Pellegrini, Francesco Nasini, Chiara Vivarelli, Marco Tavolato, Marco Mura, Francesco Parmeggiani, Giuseppe Jurman

**Affiliations:** 1 Data Science for Health Unit, Fondazione Bruno Kessler, 38123 Trento, Italy; fragni@fbk.eu (F.R.); sbovo@fbk.eu (S.B.); sona@fbk.eu (D.S.); giuseppe.jurman@fbk.eu (G.J.); 2 Department of Translational Medicine and for Romagna, University of Ferrara, 44121 Ferrara, Italy; katia.denadai@unife.it (K.D.N.); ginevragiovanna.adamo@unife.it (G.G.A.); marco.pellegrini@unife.it (M.P.); chiara.vivarelli@unife.it (C.V.); marco.mura@unife.it (M.M.); 3 ERN-EYE Network—Center Retinitis Pigmentosa of Veneto Region, Camposampiero Hospital, 35012 Padua, Italy; marco.tavolato@aulss6.veneto.it; 4 Unit of Ophthalmology, Azienda Ospedaliero Universitaria di Ferrara, 44100 Ferrara, Italy; francesco.nasini@ospfe.it; 5 Unit of Ophthalmology, Azienda ULSS Euganea di Padova, 35131 Padova, Italy; 6 King Khaled Eye Specialist Hospital, Riyadh 12211, Saudi Arabia

**Keywords:** neovascular age-related macular degeneration, optical coherence tomography, anti-VEGF drugs, artificial intelligence, machine learning, injection prediction, SHapley Additive exPlanations analysis

## Abstract

Background/Objectives: Neovascular age-related macular degeneration (nAMD) is a retinal disorder leading to irreversible central vision loss. The pro-re-nata (PRN) treatment for nAMD involves frequent intravitreal injections of anti-VEGF medications, placing a burden on patients and healthcare systems. Predicting injections needs at each monitoring session could optimize treatment outcomes and reduce unnecessary interventions. Methods: To achieve these aims, machine learning (ML) models were evaluated using different combinations of clinical variables, including retinal thickness and volume, best-corrected visual acuity, and features derived from macular optical coherence tomography (OCT). A “Leave Some Subjects Out” (LSSO) nested cross-validation approach ensured robust evaluation. Moreover, the SHapley Additive exPlanations (SHAP) analysis was employed to quantify the contribution of each feature to model predictions. Results: Results demonstrated that models incorporating both structural and functional features achieved high classification accuracy in predicting injection necessity (AUC = 0.747 ± 0.046, MCC = 0.541 ± 0.073). Moreover, the explainability analysis identified as key predictors both subretinal and intraretinal fluid, alongside central retinal thickness. Conclusions: These findings suggest that session-by-session prediction of injection needs in nAMD patients is feasible, even without processing the entire OCT image. The proposed ML framework has the potential to be integrated into routine clinical workflows, thereby optimizing nAMD therapeutic management.

## 1. Introduction

Age-related macular degeneration (AMD) is a chronic, multifactorial disorder characterized by progressive alteration of the macula, the central region of the retina responsible for high-resolution vision [1,2]. AMD is the leading cause of visual impairment and irreversible blindness among the elderly population in developed countries: about 200 million people were affected by AMD in 2020 [3], and its prevalence is projected to reach 288 million by 2040, accounting for approximately 9% of all blindness cases worldwide [4,5,6,7]. The most severe advanced stage of AMD is the exudative form, also known as neurovascular or wet AMD (nAMD), which is caused by an abnormal growth of blood vessels in the retina, leading to fluid leakage and subsequent macular degeneration. Although accounting for only 10–15% of cases globally, nAMD is responsible for up to 90% of blindness cases in AMD patients [8,9]. Appropriate evaluation procedures for the diagnosis of nAMD include an ophthalmological examination of the macula. This involves the measurement of best-corrected visual acuity (BCVA), with the Early Treatment Diabetic Retinopathy Study (ETDRS) charts representing the recommended standardized optotype for this assessment, and the evaluation of the macula using different imaging techniques, the most common being optical coherence tomography (OCT). OCT is a rapid, non-invasive, and highly repeatable imaging method, essential for both diagnosis and follow-up assessments. It enables precise measurement of retinal thickness and allows the detection of structural changes associated with disease progression or treatment response, such as retinal thickening, accumulation of subretinal and intraretinal fluid, intraretinal hyperreflective markings, and unclear boundaries of subretinal material [10,11].

Although the pathogenetic mechanism underlying AMD progression cannot be fully halted, the nAMD trajectory can be favorably influenced by intensive and sustained treatment administered over an extended period of time. The current therapeutic approach for nAMD involves intravitreal injections of anti-endothelial growth factor (anti-VEGF) agents, which are frequently employed using the pro-re-nata (PRN) regimen [12]. The PRN-related decision of injecting a patient with anti-VEGF drugs is based on follow-up visits, whose frequency depends on the level of disease activity, often involving visual acuity assessment and examination by means of macular OCT. The need for frequent and expensive intravitreal injections, coupled with the necessity of adherence to long-term treatment schedules, place a substantial burden on both patients and healthcare providers. In this context, the development of novel techniques to enhance treatment outcomes and optimize therapeutic regimens is of critical importance.

Recent advances in artificial intelligence (AI), particularly deep learning (DL), have shown promising results in analyzing OCT images for AMD-related tasks, such as distinguishing patients with AMD from those with other macular pathologies [13,14,15] or from healthy individuals [16,17,18], as well as classifying different stages of AMD [19,20,21]. Moreover, beyond diagnostic application, other studies have focused on prognostic tasks, such as predicting the progression from AMD to nAMD [22], treatment response [23], or the frequency of anti-VEGF injections required by each patient [24]—for a systematic review, see [25,26]. However, despite the high performances achieved, DL models possess inherent limitations. These include the necessity of large amounts of labeled data for effective training, significant computational resources, and generally opaque internal structures and decision-making strategies, often referred to as a “black box”. These factors might limit their suitability for deployment in clinical settings. To address these challenges, some studies have applied traditional machine learning (ML) models to quantitative features extracted from OCT images and other clinical data. A common approach for extracting relevant clinical information from OCT is the use of automated techniques. Bogunović et al. [27] developed a Random Forest model to classify patients based on their treatment requirements, using a set of clinical and quantitative spatio-temporal features derived from OCT volumes through DL algorithms. Similarly, Gallardo et al. [28] applied a ML approach to predict the long-term treatment demand of new patients using morphological tabular features automatically extracted from sequential OCT volumes. In contrast to automated segmentation techniques, other studies used clinical annotations by expert ophthalmologists on OCT images. For example, Chandra et al. [29] evaluated various ML models with the goal of predicting the required number of injections for each patient. The features assessed included retinal thickness and volume measurements, the presence and location of fluid, foveal fluid, retinal pigment epithelium (RPE) elevation, subretinal hyperreflective material, vitreomacular traction (attachment of the vitreous within the central 3 mm), and the presence of an epiretinal membrane. However, these studies primarily focused on predicting the total number of injections required, assessing disease severity [20], or evaluating treatment response [30]. None have specifically explored the potential prediction of a patient’s injection necessity on a session-by-session basis.

In the current study, the performance levels of different ML models in predicting the need for injecting a patient with anti-VEGF medication were compared, using data exclusively from the current clinical session. In addition, the impact of different combinations of input features (namely, retinal volume and thickness, best corrected visual acuity, and annotations extracted from OCT images) on classification performance was investigated. The results demonstrate that models incorporating both quantitative and structural OCT-extracted features achieved a high classification accuracy in predicting injection necessity in patients with nAMD. These findings suggest that AI-based models could be integrated into clinical workflows to optimize AMD treatment regimens, reducing the frequency of unnecessary injections.

## 2. Materials and Methods

### 2.1. Dataset

Data were collected as part of a project funded by the Italian Ministry of Health (Project code: RF-2016-02362267), aimed at investigating innovative monitoring modalities to identify the need for anti-VEGF retreatment in nAMD patients in real-life clinical settings. This study was approved by the Clinical Research Ethics Committee named CE-AVEC (Comitato Etico di Area Vasta Emilia Centro della Regione Emilia-Romagna, Italy; ethical approval code: 99/2018/Oss/AOUFe). Informed consent was obtained from all the participants or their legal guardians. All procedures followed the tenets of the Declaration of Helsinki.

Patients were recruited in a non-active phase of the disease, i.e., when no signs of exudative-hemorrhagic activity were observable. Inclusion criteria were age > 50 years, the ability and the willingness to comply with study procedures, nAMD in either treatment-naïve or previously treated patients, and a BCVA > 20/200 in the study eye. Exclusion criteria consisted of any other possible cause of neovascular maculopathy and/or the presence of ocular media opacities or other factors counteracting data collection. In the course of the selection of the study population, 11 patients were ruled out owing to the following: i. chronic persistence of exudative-hemorrhagic activity due to nAMD (7 cases); ii. BCVA reduction at a level equal to or less than 20/200 in the study eye (2 cases); iii. observation of retinal patterns indicative of myopic neovascular maculopathy (2 cases).

**Figure 1:**
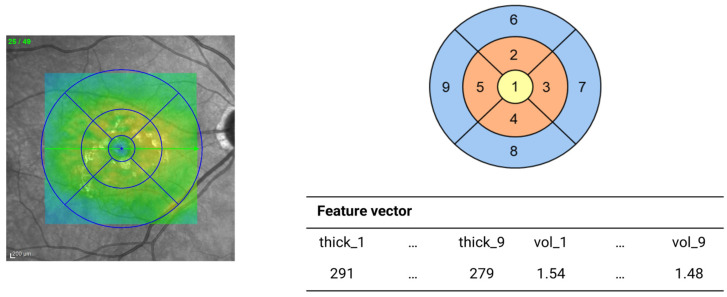
Thickness and volume OCT extraction for each ETDRS subfield. After extracting values from the Heidelberg Spectralis software, volume and thickness measurements were also combined in three concentric circles: the central circle (subfield 1; yellow), inner ring (subfields 2, 3, 4, and 5; orange), and outer ring (subfields 6, 7, 8, and 9; light blue) by averaging the corresponding values.

The selected patients underwent a comprehensive ophthalmologic examination, which included standard monitoring procedures such as the measurement of BCVA using ETDRS charts, color fundus photography (CFP), and spectral-domain optical coherence tomography (SD-OCT) using the Spectralis platform (Heidelberg Engineering Inc., Heidelberg, Germany). Based on the results of these tests, an expert ophthalmologist decided whether or not to inject the anti-VEGF drugs into the target eye. This therapeutic decision after routine monitoring visits was used as the gold-standard reference. In particular, according to the PRN retreatment criteria of the National Institute for Health and Care Excellence (NICE guideline NG82 available at https://www.nice.org.uk/guidance/ng82—accessed on 15 November 2024), an intravitreal injection of the anti-VEGF drug was scheduled only if signs of active wAMD were present, such as a i. decrease in BCVA related to exudative-hemorrhagic activity; ii. increase in OCT-evaluated macular fluids, cysts, and/or detachments due to choroidal neovascularization; or iii. occurrence of new hemorrhagic events secondary to the maculopathy. The study design also defined the timing of intervention following a PRN regimen. Visits were scheduled every 30 ± 15 days, with treatments administered within 7 ± 3 days following each visit, over a maximum time window of 18 months. Moreover, intra-patient factors, potentially affecting ophthalmic exams, were assessed at the baseline and subsequently every 3 months. A total of 557 sessions with 47 patients at the Eye Clinic of Ferrara University Hospital (Italy) were considered for the following analyses. Due to some anomalies in the collected measurements, a patient was removed from the dataset, leading to a total of 540 experimental sessions. For each session, three sets of variables were considered. The first category included quantitative clinical variables extracted from OCT images and annotated by experienced clinicians, such as the presence of subretinal and intraretinal fluid, intraretinal cysts and/or macular edema, the detachment of neuroepithelium (NE) and/or of retinal pigment epithelium (RPE), and nerve fiber layer assessment. The second set encompassed mean macular thickness (µm) and volume (mm^3^) measurements, generated by the Heidelberg Spectralis software (version 6.9.5) during the OCT scanning procedure, and relative to the 9 subfields of the ETDRS grid. These subfields were further combined into three concentric zones: central circle (1 mm diameter), inner ring (3 mm diameter), and outer ring (6 mm diameter; see Figure 1), by averaging the corresponding values. The third variables set comprised the standardized BCVA, measured by the ETDRS chart and expressed in logMAR.

### 2.2. Preprocessing

**Figure 2:**
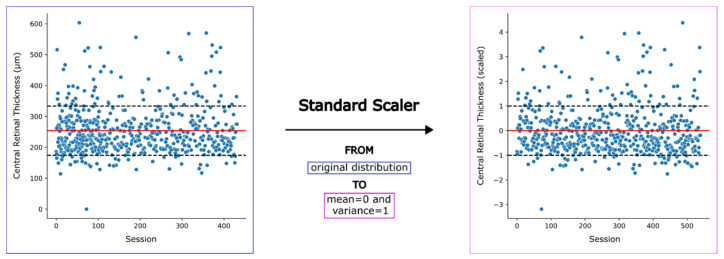
Preprocessing of numerical features using a standard scaler with z-score normalization. The left plot shows the original distribution of feature values for a sample numerical feature (i.e., central retinal thickness) across patient sessions. The right plot displays the same feature after normalization using a standard scaler: the data distribution is transformed to have a mean of 0 and a variance of 1. In both plots, the red solid line represents the mean, while the black dashed lines indicate one standard deviation above and below the mean. Standard scaling was applied to all numerical predictors to ensure consistency in model training.

The outcome variable was encoded by binary labels assigning the positive tag to sessions in which the clinical decision of injecting the patient was made, and zero otherwise. All sessions were treated as independent data points. Before training the models, numerical predictors were normalized between 0 and 1 using a standard scaler (see Figure 2). Additionally, the macular edema and the nerve fiber layer assessment variables were excluded due to their low variance. The overall set of variables used for model training along with their characteristics are shown in Table 1.

### 2.3. Machine Learning Pipeline

First, to identify the model that would best perform on our dataset, five different ML algorithms were selected, namely support vector (SVC), Random Forest (RF), Extra Trees, Gradient Boost, and Extreme Gradient Boosting (XGB) classifiers. The selected models were then evaluated on different combinations of input data, to assess the contributions of various features. All subsets included the volume and thickness of each ETDRS subfield, which comprised the first feature set (C1). The second combination (C2) included BCVA in addition to volume and thickness data. The third set (C3) added clinical annotations (as detailed in Table 1) to C1 variables, while the fourth set (C4) combined the volume and thickness, BVCA, and clinical annotations.

**Figure 3:**
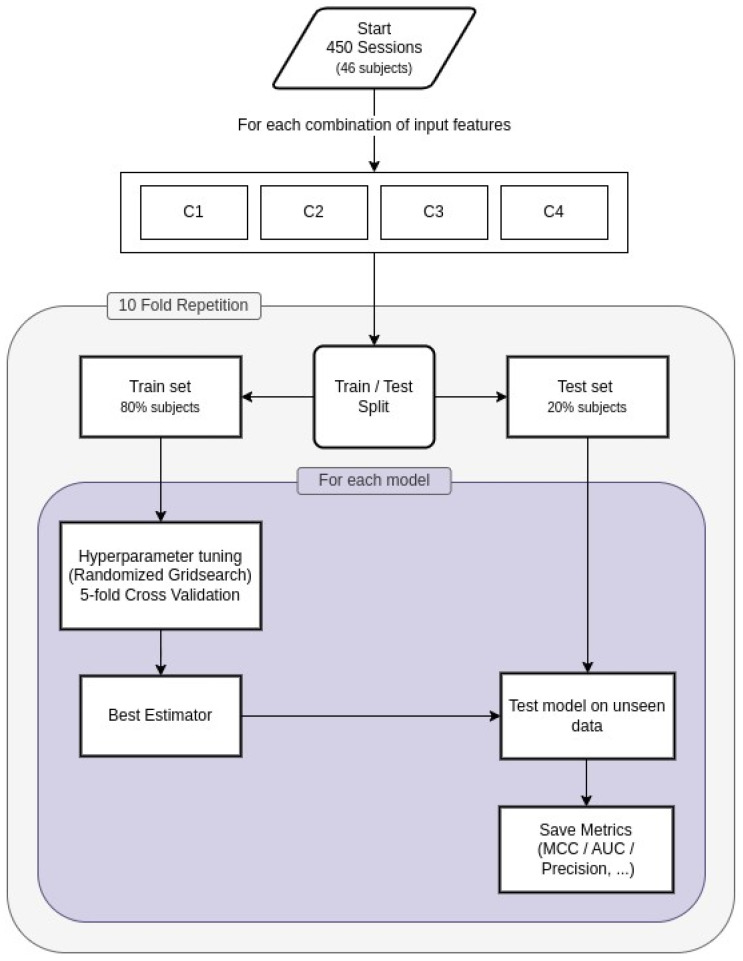
Schematic depiction of the “Leave Some Subjects Out” (LSSO) cross-validation approach. For each input combination (i.e., C1, C2, C3, C4), the dataset was randomly divided into training (sessions pertaining to 80% of the patients) and test (sessions pertaining to 20% of the patients) sets. Each model was then optimized by means of a randomized grid search on the training set, and tested on the test set. This process was repeated 10 times, and the results were averaged to select the best-performing model for each combination of input parameters.

The performances of the aforementioned models were evaluated using a “Leave Some Subjects Out” (LSSO) cross-validation approach. This consists of systematically selecting all sessions pertaining to a random subset of subjects during training, and testing the resulting model on those. This procedure makes it possible to account for subject-specific variations, and it helps in understanding whether the model can generalize to new subjects’ sessions that were not seen during training. For testing, all sessions pertaining to 9 patients (20% of the total number of subjects) were chosen as the “left out” data, while the remaining sessions from 37 patients (80% of the sample) were used for training. This process was repeated 10 times, each time selecting a different set of subjects for testing and training. During the training phase, the hyperparameters of each model were optimized by means of a randomized grid search approach. This method evaluates multiple combinations of hyperparameters and selects those achieving higher performances for the current cross-validation fold. The hyperparameter tuning during the training step was finalized to maximize the Matthews correlation coefficient (MCC) score. The MCC score is a metric ranging from −1 to +1, where +1 indicates a perfect model, 0 represents a random prediction, and −1 a poor model. This metric was selected as it takes into account all four components of the confusion matrix (true positives, true negatives, false positives, and false negatives), providing a more comprehensive evaluation compared to other metrics [31,32,33]. This process was repeated for each of the four combinations of input data, and the performance metrics were then averaged across the 10 train–test splits for each combination. The best-performing model for each combination of input features was selected for comparison. The pipeline structure is shown in Figure 3.

To assess the predictive performance levels of the models, several metrics were considered, such as recall, accuracy, F1 score, area under the receiver operating characteristic curve (ROC AUC), and MCC.

### 2.4. Predictive Model Interpretability

To increase the interpretability of our machine learning analyses, the SHapley Additive exPlanations (SHAP) method was applied to the best-performing model [34]. This method has gained significant attention in the machine learning community due to its interpretability, enabling users to understand complex model behaviors and make informed decisions. The SHAP method allows for the inspection of the predictive power of individual variables by highlighting how each feature impacts the final prediction, both at the instance level and across the whole population. For each iteration, SHAP values were calculated for the training set data based on the fitted model. To evaluate the overall effect of the features, these values were then combined into a single Beeswarm plot.

## 3. Results

In the investigative context aimed at developing a system that could assist clinicians in the yes/no decision about intravitreal administration of anti-VEGF drugs to patients with nAMD, we studied the performance of various machine learning (ML) algorithms using real-life data collected during a strict PRN therapeutic regimen.

### 3.1. Predictive Performance

**Figure 4:**
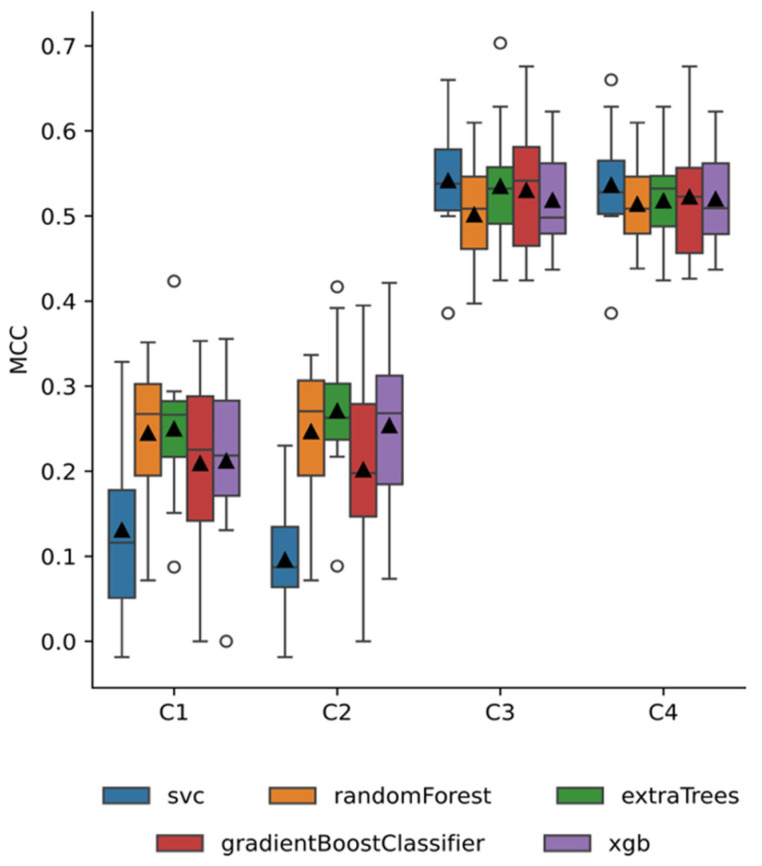
Boxplots displaying the distribution of MCC scores for each type of machine learning model (blue: SVC, orange: Random Forest, green: Extra Trees Classifier, red: Gradient Boost Classifier, purple: Extreme Gradient Boost Classifier) and input features combinations (C1: volume and thickness of each ETDRS subfield, C2: C1 and BCVA, C3: C1 and clinical annotations, C4: C1, BCVA, and clinical annotations), across the ten iterations of the LSSO procedure. Black lines represent the median, black triangles the mean, whiskers 1.5× the interquartile range and circles data points falling beyond 1.5× the interquartile range.

As an initial step, we aimed at finding which model performed the best for each combination of input features. For feature sets (i.e., C1, C2, C3, and C4), we evaluated the performance of different ML models, i.e., ETC, RF, GB, XGB, and SVC classifiers. Models were evaluated using several performance metrics, including ROC AUC, accuracy, recall, F1, and MCC. A summary of classification results for each combination of input features is presented in Figure 4.

### 3.2. Selection of Most Informative Input Features

**Figure 5:**
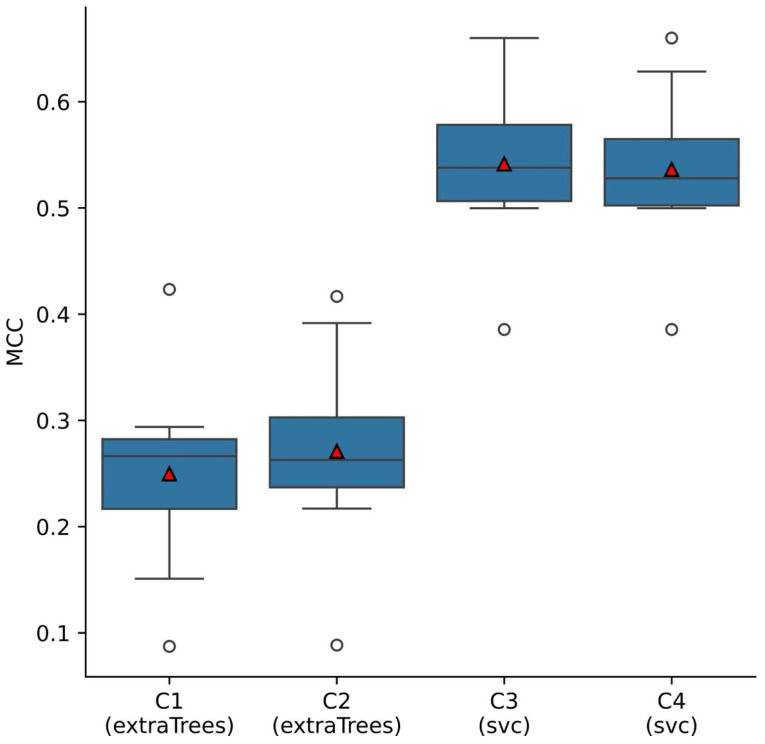
Boxplots displaying the distribution of MCC scores for each type of machine learning model (Extra Trees Classifier, SVC) and input feature combinations (C1: volume and thickness of each ETDRS subfield, C2: C1 and BCVA, C3: C1 and clinical annotations, C4: C1, BCVA, and clinical annotations), across the ten iterations of the LSSO procedure. Black lines represent the median, red triangles the mean, whiskers 1.5× the interquartile range, and circles data points falling beyond 1.5× the interquartile range.

After determining the optimal performance for each combination of input features, the next step was identifying which combination yielded the best overall performance. Models trained on input feature combinations that included clinical annotations exhibited higher median MCC scores (C3: MCC = 0.541 ± 0.07; C4: MCC = 0.536 ± 0.07) compared to those using only volumes, thickness, and BCVA (i.e., C1: mean MCC = 0.225 ± 0.11; C3: mean MCC = 0.231 ± 0.11). Overall, the model achieving the best performance was the SVC trained with the third combination of input features (C3: volumes, thickness and clinical annotations), attaining a mean MCC score of 0.5415 ± 0.073. As illustrated in Figure 5, this algorithm had a more contained spread and higher median score compared to the other considered models.

**Figure 6:**
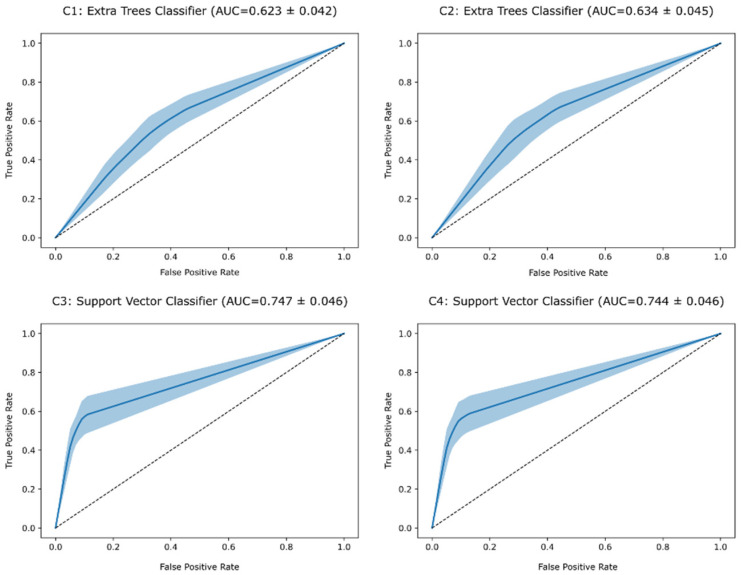
ROC AUC curves for the best-performing model for each combination of input parameters (C1, C2, C3, and C4). The solid blue line represents the average ROC AUC across the 10 iterations of the “Leave-Some-Subjects-Out” (LSSO) cross-validation procedure, while the shaded area indicates the standard deviation. The dotted black line represents the ROC curve of a random classifier.

A comprehensive summary of the results is provided in Table 2, and the corresponding ROC AUC curves are displayed in Figure 6.

### 3.3. Model Interpretability

**Figure 7:**
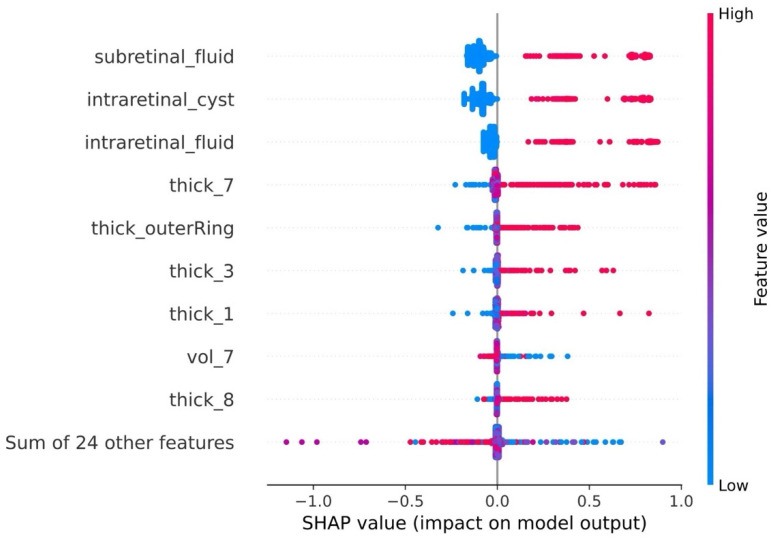
SHAP Beeswarm plot listing the top 9 features impacting model outputs. Each point represents a SHAP value for a feature and an individual observation. The blue color represents low values for a variable, while red indicates high values. A higher SHAP value indicates a positive influence on the model’s prediction of the necessity to administer anti-VEGF medications to the patient.

To further explain the predictive performance of the best model (i.e., SVC trained on input feature set C3), a saliency analysis using SHAP was performed. Figure 7 illustrates the top nine variables with the highest impact on model prediction.

The necessity of intravitreal treatment with anti-VEGF medications is associated with both clinical annotations and OCT-derived variables. Regarding the former, the presence of subretinal fluid, intraretinal cysts, and intraretinal fluid has a positive influence on the target outcome. For physiological variables, an increase in macular thickness within the outer ring, or in sectors 7, 3, and 1, and a decrease in macular volume were more closely associated with the necessity of treating patients with anti-VEGF medications.

## 4. Discussion

In this study, the possibility of using artificial intelligence to predict the need for anti- VEGF injections in patients with nAMD was tested, based on data from individual clinical sessions. To determine which data types might be more suitable for this task, different combinations of input features, including retinal volume and thickness information, BCVA, and quantitative clinical annotations extracted from OCT images, were selected. Moreover, the performance levels of different ML algorithms were compared using a robust nested cross-validation approach to ensure reliable results across train–test splits. The findings indicated that models incorporating clinical annotations outperformed those based solely on retinal volume and thickness measurements, and that adding BCVA values did not improve prediction performances in either case. Overall, the model with the highest predictive power was an SVC, achieving an MCC score of 0.5415 ± 0.07. Feature importance analysis revealed that clinical annotation, specifically the presence of subretinal and intraretinal fluid, alongside OCT-derived features like retinal thickness, were key predictors for the model.

The results obtained align with previous studies, which highlighted the fundamental role of OCT-derived clinical annotations in enhancing the predictive power of ML models. For instance, Chandra et al. [29] employed quantitative and qualitative evaluation of lesion characteristics extrapolated from OCT images to predict the number of anti-VEGF injections required by each patient. Their results emphasized the presence of intraretinal and subretinal fluid and sub-retinal pigment epithelium (RPE), along with baseline lesion characteristics, as the most influential features for model prediction. In a similar study, Gallardo et al. [28] aimed at stratifying patients based on treatment demand by incorporating retinal volume and thickness measurements alongside clinical annotations of morphological retinal features automatically extracted from OCT volumes. Their feature importance analysis highlighted as most representative variables the presence of subretinal (SRF) and intraretinal fluid (IRF).

Interestingly, in the current study the inclusion of BCVA, whether combined with retinal thickness and volume measurements or in conjunction with OCT quantitative features, did not improve model performances. To date, the contribution of BCVA in ML applications for nAMD remains unclear. Bogunović et al. [27] reported a limited impact of BCVA on accuracy when predicting anti-VEGF treatment requirements in nAMD patients. In contrast, other studies have shown that BCVA plays a crucial role in predicting visual acuity outcomes at 9 [35] and 12 months [36] after anti-VEGF treatment. This discrepancy might be linked to the specific aim of the studies, suggesting a more relevant role of BCVA in analyses focused on long-term visual outcomes.

To explain the behavior of their models, previously cited papers applied feature importance techniques that explored the magnitude of each feature’s contribution to model performance, but did not provide insights relative to the directionality of these effects. To address this limitation, in the current study, a SHAP analysis was performed. This approach provides a clear understanding of how feature values influence model predictions, and thus might act as protective or a risk factor for a specific task. The SHAP analysis identified OCT-derived features, such as the presence of subretinal fluid, intraretinal cysts, and intraretinal fluid, as the most influential predictors for determining the need for injections. Additionally, increased retinal thickness, particularly in the central region (ETDRS subfield 1) near the fovea, and in regions adjacent to the optic disk (zones 3–7), was associated with a higher likelihood of requiring treatment.

By narrowing the prediction window to individual sessions, we aim to provide a more actionable framework that might support clinicians in optimizing treatment regimens and potentially reduce unnecessary injections. Most patients with AMD require approximately 7−8 injections in the first 12 months to effectively manage the wet form of the disease [37,38], with a reduced frequency generally needed in the subsequent years. This places a significant burden on physicians, staff, patients, and caregivers [39], as well as a substantial economic strain on the healthcare system. Compared to previous studies that primarily focused on predicting disease progression [22], treatment response [23], or the frequency of anti-VEGF injections required by each patient [24], we propose a more granular approach, that might be specifically relevant in normal clinical practice, during which the decision to schedule a new injection (pro-re-nata regimen) or to modify the injection timing (treat-and-extend regimen) is made at each visit [40,41]. In real-world clinical settings, an automated model could serve as a valuable complementary support system for both less experienced clinicians and experts with high workloads, providing a preliminary indication of the need for anti-VEGF injections. This could potentially reduce the time to treatment and enhance decision-making reliability. The strength of an automated ML model in this context lies in its ability to provide consistent data-driven recommendations. As a result, this framework could also potentially be integrated into telemedicine, helping the decentralization of AMD management by separating data collection from data interpretation. Orthoptists could collect clinical data in community settings, enabling a broader patient outreach. The data would then be sent to a central reading center where ophthalmologists, supported by a ML model, would evaluate and annotate OCT images, and generate predictions to inform treatment decisions. The proposed model would be well-suited for this type of setting, as it does not require significant computational resources, is easy to deploy, and can predict outcomes for individual sessions without the need for additional contextual information.

However, several limitations of the current study should be acknowledged. First, the relatively small sample size and the use of simpler machine learning algorithms might have constrained the performance of the presented model. To address these limitations, future works might explore the application of DL approaches, which, in light of their ability to capture more complex, non-linear relationships in data, might lead to improved classification performances. Given the longitudinal nature of session-based clinical data, algorithms capable of modeling temporal dependencies, such as Recurrent Neural Networks (RNNs) or transformers [42], could be explored (see [43,44] for potential applications). Leveraging the computational power of these models would require a larger dataset, encompassing a greater number of patients and clinical sessions, to achieve more robust results. To this end, recent research has focused on augmenting existing datasets through synthetic data generation (for a systematic review, see [45,46]), which involves creating artificial observations that mimic the statistical properties and patterns of real data. Common methods for generating synthetic data include deep learning models, such as Generative Adversarial Networks (GANs) or Variational Autoencoders (VAEs), whose application could be considered in future studies to increase data availability and enhance model performance. In addition, quantitative OCT annotations were performed by an expert ophthalmologist after the manual inspection of each individual image, which is both time-consuming and costly and thus might limit the scalability in routine clinical settings. A potential solution to this challenge would be the use of automated DL-based tools for feature extraction from OCT imaging, as several studies reported their efficacy in producing reliable quantitative annotations (for a review, see [25]). Finally, the presented approach did not explore multimodal integration of different data sources, which could potentially enhance classification performances. Several studies have highlighted the benefits of combining information from different data sources (e.g., images and clinical data) for training AI models [47]. Future work might investigate the integration of multiple data sources to better reflect the complexity of decision-making in the therapeutic management of nAMD.

## Figures and Tables

**Table 1:** List of selected features for the algorithm training process. For each variable, a data type description, mean, and standard deviation are reported.

Datum Type	Feature Name	Variable Type (Levels)	Value (Mean ± SD or Numbers)
OCT-derived information	Volume (sectors 1–9)	Numerical	0.9078 mm^3^ ± 0.5674
	Volumetric map	Numerical	292.05 mm^3^ ± 76.6557
	Thickness (sectors 1–9)	Numerical	301.1327 µm ± 67.1518
	Central retinal thickness	Numerical	254.0352 µm ± 79.7335
OCT-derived annotation	Subretinal fluid	Categorical (0;1)	0 (*n* = 471); 1 (*n* = 69)
	Intraretinal fluid	Categorical (0;1)	0 (*n* = 502); 1 (*n* = 38)
	Intraretinal cyst	Categorical (0;1)	0 (*n* = 476); 1 (*n* = 64)
	NE detachment	Categorical (0;1)	0 (*n* = 522); 1 (*n* = 18)
	RPE detachment	Categorical (0;1)	0 (*n* = 479); 1 (*n* = 61)
Visual function	BCVA	Numerical	0.0895 logMAR ± 0.127

SD = standard deviation; OCT = optical coherence tomography; NE = neuroepithelium; RPE = retinal pigment epithelium; BCVA = best-corrected visual acuity.

**Table 2:** Evaluation metrics for the LSSO cross-validation analysis. For each subset of input features (i.e., C1, C2, C3, C4), the metrics of the model with the highest mean MCC are reported. Comprehensive tables detailing the list of optimized hyperparameters, the corresponding selected values, and the metrics of all models trained with the different combinations of input features can be found in Appendix A, Table A1, Table A2 and Table A3.

Input Feature Combination	ROC AUC	Accuracy	F1 Score	Recall	MCC
C1 (ETC)	0.623 ± 0.042	0.626 ± 0.045	0.579 ± 0.055	0.596 ± 0.108	0.25 ± 0.085
C2 (ETC)	0.634 ± 0.045	0.638 ± 0.046	0.59 ± 0.064	0.607 ± 0.116	0.271 ± 0.087
**C3 (SVC)**	**0.747 ± 0.046**	**0.77 ± 0.034**	**0.675 ± 0.081**	**0.564 ± 0.106**	**0.541 ± 0.073**
C4 (SVC)	0.744 ± 0.046	0.768 ± 0.034	0.672 ± 0.08	0.56 ± 0.105	0.536 ± 0.072

ROC AUC = area under the receiver operating characteristic curve; MCC = Matthews correlation coefficient. The best-performing model overall is highlighted in bold.

## Data Availability

Data are not available due to privacy restrictions. The authors take responsibility for the integrity of the data and the accuracy of the data analysis.

## Appendix A

**Table A1:** Evaluation metrics for the “Leave Some Subjects Out” (LSSO) cross-validation analysis. For each subset of the available features, all the metrics for the 5 models are shown. C1: volume and thickness of each ETDRS sector, C2: C1 + BCVA, C3: C1 + clinical annotations, C4: C1 + BCVA + clinical annotations.

C	Model	ROC AUC	Accuracy	F1	Recall	MCC
C1	SVC	0.564 ± 0.052	0.573 ± 0.057	0.504 ± 0.075	0.509 ± 0.122	0.131 ± 0.103
C1	RFC	0.62 ± 0.041	0.624 ± 0.044	0.57 ± 0.063	0.584 ± 0.126	0.245 ± 0.08
C1	ETC	0.623 ± 0.042	0.626 ± 0.045	0.579 ± 0.055	0.596 ± 0.108	0.25 ± 0.085
C1	GBC	0.601 ± 0.054	0.612 ± 0.058	0.532 ± 0.082	0.517 ± 0.126	0.209 ± 0.11
C1	XGBC	0.602 ± 0.044	0.605 ± 0.055	0.543 ± 0.082	0.557 ± 0.149	0.212 ± 0.095
C2	SVC	0.547 ± 0.032	0.556 ± 0.034	0.474 ± 0.072	0.472 ± 0.12	0.096 ± 0.065
C2	RFC	0.621 ± 0.039	0.628 ± 0.043	0.564 ± 0.057	0.56 ± 0.109	0.246 ± 0.078
C2	ETC	0.634 ± 0.045	0.638 ± 0.046	0.59 ± 0.064	0.607 ± 0.116	0.271 ± 0.087
C2	GBC	0.598 ± 0.057	0.612 ± 0.056	0.512 ± 0.115	0.492 ± 0.156	0.202 ± 0.11
C2	XGBC	0.62 ± 0.049	0.633 ± 0.055	0.546 ± 0.082	0.523 ± 0.135	0.254 ± 0.099
C3	SVC	0.747 ± 0.046	0.77 ± 0.034	0.675 ± 0.081	0.564 ± 0.106	0.541 ± 0.073
C3	RFC	0.734 ± 0.031	0.748 ± 0.036	0.683 ± 0.053	0.635 ± 0.137	0.501 ± 0.066
C3	ETC	0.742 ± 0.048	0.764 ± 0.042	0.67 ± 0.083	0.569 ± 0.135	0.535 ± 0.08
C3	GBC	0.75 ± 0.047	0.764 ± 0.038	0.696 ± 0.082	0.643 ± 0.145	0.53 ± 0.079
C3	XGBC	0.749 ± 0.033	0.761 ± 0.03	0.702 ± 0.058	0.659 ± 0.116	0.518 ± 0.057
C4	SVC	0.744 ± 0.046	0.768 ± 0.034	0.672 ± 0.08	0.56 ± 0.105	0.536 ± 0.072
C4	RFC	0.736 ± 0.03	0.754 ± 0.032	0.68 ± 0.06	0.619 ± 0.15	0.514 ± 0.051
C4	ETC	0.733 ± 0.036	0.756 ± 0.031	0.662 ± 0.069	0.563 ± 0.124	0.518 ± 0.058
C4	GBC	0.746 ± 0.043	0.76 ± 0.04	0.694 ± 0.071	0.642 ± 0.133	0.522 ± 0.076
C4	XGBC	0.752 ± 0.029	0.763 ± 0.03	0.712 ± 0.046	0.678 ± 0.095	0.52 ± 0.057

C = combination; ROC AUC = area under the receiver operating characteristic curve; MCC = Matthews correlation coefficient; SVC = support vector classifier; RFC = Random Forest Classifier; ETC = Extra Trees Classifier; GBC = Gradient Boost Classifier; XGBC = Extreme Gradient Boosting Classifier.

**Table A2:** List of hyperparameters optimized for each algorithm (i.e., SVC, Random Forest, Extra Trees Classifier, Gradient Boost, and XGBoost) during the “Leave Some Subjects Out” (LSSO) cross-validation procedure, along with the specific ranges of values explored.

Model	Hyperparameter	Range/Values
SVC	C	Exponential distribution (λ = 0.01)
	kernel	[‘rbf’]
	gamma	Exponential distribution (λ = 10)
Random Forest	n_estimators	[50, 51, …, 499]
	max_depth	[10, 20, 30, 40, 50, None]
	max_features	[‘log2’, ‘sqrt’, None]
	min_samples_split	Uniform distribution [0, 1]
	min_samples_leaf	Uniform distribution [0, 1]
	bootstrap	[True, False]
	criterion	[‘gini’, ‘entropy’]
Extra Trees	n_estimators	[50, 51, …, 499]
	max_depth	[1, 2, …, 49]
	max_features	[1, 2, …, X.shape-1]
	class_weight	[‘balanced’]
Gradient Boost	learning_rate	Uniform distribution [0.01, 0.5]
	n_estimators	[50, 51, …, 499]
	subsample	[1.0]
	max_depth	[1, 3, 5, 10, 20, 30, 40, 50, None]
	min_samples_split	Uniform distribution [0, 1]
	min_samples_leaf	Uniform distribution [0, 1]
	max_features	[None, ‘sqrt’, ‘log2’]
	loss	[‘log_loss’, ‘exponential’]
XGBoost	learning_rate	Uniform distribution [0.001, 0.5]
	subsample	[0.5, 0.7, 1]
	n_estimators	[50, 51, …, 499]
	max_depth	[1, 3, 5, 10, 20, 30, 40, 50, None]
	gamma	Uniform distribution [0, 10]

ETC = Extra Trees Classifier; SVC = support vector classifier; C = regularization parameter; kernel = kernel type; gamma = kernel coefficient; n_estimators = number of trees; max_depth = maximum depth of the tree; max_features = maximum features to consider; min_sample_split = minimum number of samples required to split an internal node; min_samples_leaf = minimum number of samples required to be at a leaf node; bootstrap = bootstrap applied; criterion = function to measure the quality of a split; class_weight = weight associated with classes; learning_rate = learning rate value; subsample = fraction of samples to be used for fitting individual base learners; loss = loss function to be optimized.

**Table A3:** Hyperparameters selected for the best-performing models and their corresponding input data combinations (C1, C2, C3, and C4), optimized across the 10 iterations of the “Leave-Some-Subjects-Out” (LSSO) cross-validation procedure.

Model	Hyperparameter	Value
C1: ETC	n_estimators	235
	max_features	23
	max_depth	7
	class_weight	‘balanced’
C2: ETC	n_estimators	123
	max_features	7
	max_depth	8
	class_weight	‘balanced’
C3: SVC	C	82.6
	gamma	1.67 × 10^−4^
	kernel	‘rbf’
C4: SVC	C	82.6
	gamma	1.67 × 10^−4^
	kernel	‘rbf’

C1, C2, C3, C4 = input features combinations; ETC = extra trees classifier; SVC = support vector classifier; n_estimators = number of trees; max_features = maximum features to consider; max_depth = maximum depth of the tree; class_weight = weight associated with classes; C = regularization parameter; gamma = kernel coefficient; kernel = kernel type.
